# Optimized Spatial Priorities for Biodiversity Conservation in China: A Systematic Conservation Planning Perspective

**DOI:** 10.1371/journal.pone.0103783

**Published:** 2014-07-29

**Authors:** Ruidong Wu, Yongcheng Long, George P. Malanson, Paul A. Garber, Shuang Zhang, Diqiang Li, Peng Zhao, Longzhu Wang, Hairui Duo

**Affiliations:** 1 Institute of International Rivers and Eco-security, Yunnan University, Kunming, Yunnan, China; 2 Yunnan Key Laboratory of International Rivers and Transboundary Eco-security, Yunnan University, Kunming, Yunnan, China; 3 The Nature Conservancy China Program, Kunming, Yunnan, China; 4 Department of Geography, The University of Iowa, Iowa City, Iowa, United States of America; 5 Department of Anthropology, University of Illinois, Urbana, Illinois, United States of America; 6 Institute of Forest Ecology, Environment, and Protection, Chinese Academy of Forestry, Beijing, China; 7 School of Nature Reserve, Beijing Forestry University, Beijing, China; Fondazione Edmund Mach, Research and Innovation Centre, Italy

## Abstract

By addressing several key features overlooked in previous studies, i.e. human disturbance, integration of ecosystem- and species-level conservation features, and principles of complementarity and representativeness, we present the first national-scale systematic conservation planning for China to determine the optimized spatial priorities for biodiversity conservation. We compiled a spatial database on the distributions of ecosystem- and species-level conservation features, and modeled a human disturbance index (HDI) by aggregating information using several socioeconomic proxies. We ran Marxan with two scenarios (HDI-ignored and HDI-considered) to investigate the effects of human disturbance, and explored the geographic patterns of the optimized spatial conservation priorities. Compared to when HDI was ignored, the HDI-considered scenario resulted in (1) a marked reduction (∼9%) in the total HDI score and a slight increase (∼7%) in the total area of the portfolio of priority units, (2) a significant increase (∼43%) in the total irreplaceable area and (3) more irreplaceable units being identified in almost all environmental zones and highly-disturbed provinces. Thus the inclusion of human disturbance is essential for cost-effective priority-setting. Attention should be targeted to the areas that are characterized as moderately-disturbed, <2,000 m in altitude, and/or intermediately- to extremely-rugged in terrain to identify potentially important regions for implementing cost-effective conservation. We delineated 23 primary large-scale priority areas that are significant for conserving China's biodiversity, but those isolated priority units in disturbed regions are in more urgent need of conservation actions so as to prevent immediate and severe biodiversity loss. This study presents a spatially optimized national-scale portfolio of conservation priorities – effectively representing the overall biodiversity of China while minimizing conflicts with economic development. Our results offer critical insights for current conservation and strategic land-use planning in China. The approach is transferable and easy to implement by end-users, and applicable for national- and local-scale systematic conservation prioritization practices.

## Introduction

Anthropogenic effects have resulted in the loss of biodiversity at an unprecedented rate, while resources for biodiversity conservation remain constrained in terms of both human and financial capacity [1]. That is why the systematic planning of priority areas is crucial to achieve the most cost-effective conservation, such as identifying large-scale biodiversity hotspots or assembling fine-resolution portfolios of conservation priorities [2]–[4]. In the last two decades, systematic conservation planning (SCP) has emerged as an effective approach for identifying conservation priorities [3]–[6]. SCP aims to identify a network of priority areas so as to effectively achieve explicit conservation goals in terms of representing the full range of biodiversity and sustaining their long-term survival [5]. Efficient conservation priorities can be identified through an optimized planning algorithm for meeting conservation goals at the minimum land area or other costs (e.g., land prices, management and opportunity costs [7]). SCP provides an operational framework for minimizing land-use conflicts between conserving natural environments and economic development, and thus increase the likelihood of implementing the proposed conservation actions [3], [6]. Here, we present the first national-scale SCP study for China to determine the optimized spatial priorities for biodiversity conservation.

China – one of the world's “megadiversity countries” – is home to many globally valued conservation priorities [2]. However, China's biodiversity is under severe threat due to the increasing pressure resulting from the country's historically unprecedented economic growth [8]. Meanwhile, China's conservation investment is considerably lower compared to developed and other developing countries [9]. Thus, the systematic conservation priority-setting has been emphasized in China during last two decades [8], [10]–[11].

During this period, China has developed several templates of national-scale conservation priorities, which were based principally on the species (e.g., endemic, threatened, and/or other indicator species) richness patterns as well as expert judgments (e.g., [10], [12]–[15]). These templates are crucial in guiding China's national-level conservation decisions; however, we think there are several critical limitations in previous priority-setting studies.

First, the effects of human disturbance are not incorporated in previous studies, whereas we believe explicit inclusion of human disturbance in priority-setting can minimize land-use conflicts and lower costs for meeting conservation goals [3], [6]. Second, the scoring procedure in these studies is inefficient for achieving the goal of full representation of all biodiversity targets [16], i.e. the goal for representativeness – one of the core principles for designing an efficient reserve system [5]. The current scoring procedure requires a greater amount of land (and increases other costs) to achieve the same conservation goals and these greater demands are unlikely to get support from local authorities. Third, a study designed to systematically integrate conservation features at both ecosystem- and species-level is still lacking, as the conservation features used in previous studies are either species or ecosystem based. By incorporating biodiversity features from multiple organization levels, the resulting portfolio of conservation priorities is more efficient in representing the full range of biodiversity concerns and in maintaining the ecological integrity of ecosystems [5], [16]. SCP can overcome this inefficiency in scoring procedure by employing the principle of between-site complementarity that serves to boost the efficient representation of all biodiversity targets, and provide mechanisms for integrating human disturbance and conservation features at multiple organization levels [17].

This study aims to determine the optimized national-scale spatial priorities in China and to ensure effectively fulfilling biodiversity conservation goals given the constraints of human disturbance by implementing a SCP approach. Taiwan, Hong Kong and Macao are not included in our analysis due to lack of required information. Specifically, we are trying to address two questions: (1) How will the inclusion of human disturbance affect the result of conservation priority-setting? (2) What are the geographical patterns of the optimized conservation priorities in China? In this analysis, we integrated human disturbance, conservation features at both the ecosystem- and species-level, and the principles of complementarity and representativeness. We used the software Marxan [17] to determine each unit's conservation value and to identify priorities with regular hexagons (100 km^2^ per cell) as the planning units. We investigated the effects of human disturbance using two Marxan scenarios – a disturbance ignored scenario and a disturbance considered scenario. For the second scenario, human disturbance was included as a penalty function by aggregating information on several socioeconomic proxies as an index layer. We then explored the spatial patterns of the priority units, irreplaceable areas, and primary large-scale priority areas (i.e., the large clustered regions of high-conservation-value units). The analysis is limited to the data available at national-scale and applicable resolution, and misses some variability within the range of human disturbances. We believe this study is applicable to national- and local-scale conservation and other sustainable land-use planning for systematically evaluating each site's conservation value and identifying spatially optimized priority areas.

## Methods

### Conservation Features Mapping

Considering the complexity of biodiversity and severe lack of detailed spatial distribution data, surrogates (e.g., endangered/endemic species, key habitat types and environmental features) are often used in conservation planning [3], [7], [16], [18]. Integrating conservation features from multiple levels can ensure the efficient representation of biodiversity [5] and compensate for limitations in the data [16]. In this analysis, we used both ecosystem- and species-level features as the surrogates.

The ecosystem-level features included were: (1) priority natural ecosystems as defined by Li, Song & Ouyang [13], including 129 natural ecosystems of forests, grasslands, meadows, deserts and wetlands, and (2) natural vegetation types derived from the national 1: 1,000,000 vegetation map, including 559 natural vegetation formations [19]. This study considered wetlands and lakes (in the priority natural ecosystems and natural vegetation types), but data on aquatic systems and species was lacking. We expected that China's key ecological elements, processes and services were covered with priority natural ecosystems and that basic habitat types were represented by finer-scale classifications of natural vegetation types. The species-level features were endangered species of plants, mammals, and birds. Endangered mammals and birds were identified according to China's “National List of Key Protected Wildlife” and the IUCN Red List Categories of critically endangered, endangered and vulnerable species [20]. Endangered plants were defined in the “China Plant Red Data Book: Endangered and Rare Plants” [21].

Previous studies often use county-level species distribution data derived from the published literatures [14]–[15], while our analysis was performed using a finer-scale resolution. For plants and mammals, we mapped each species' geographic range by combining its distribution data for counties, preferred habitat types and elevation range. For a bird species, the range was derived by intersecting only counties and habitat types, because knowledge of the altitude distribution of most avian species is lacking. This mapping process included: (1) collecting each species' attribute information, i.e. species name, taxonomy, endangered category, distribution across counties, preferred habitat types, and elevation range, (2) mapping each species' distributions across counties, habitat types and elevation range, respectively, and (3) identifying the overlap region among these distribution layers as each species' current range.

We collected the attribute information using the following resources. For plants, we used “National Key Protected Wild Plant Resources Survey” [22] as the primary source and other supplementary sources including “Subject Database of China Plants” [23], “China Species Information Services” [24] and “China Plant Red Data Book: Endangered and Rare Plants” [21]. For mammals and birds, we used “National Key Terrestrial Wildlife Resources Survey” [25] as the primary source and other supplementary sources including “Database of Fauna Sinica” [26], “Distributions of China Mammal Species” [27] and “China Red Data Book of Endangered Animals: Mammals” [28].

The datasets on county boundaries and habitat types were derived from the national 1∶ 1,000,000 geographic databases and the national 1∶ 1,000,000 vegetation map [19], respectively. The elevation range for each species was extracted from the Shuttle Radar Topography Mission (SRTM) 90 m Digital Elevation Model (DEM) [29]. We mapped the species' geographic ranges for 373 plant, 115 mammal and 81 bird species.

### Human Disturbance Index Mapping

**Figure 1:**
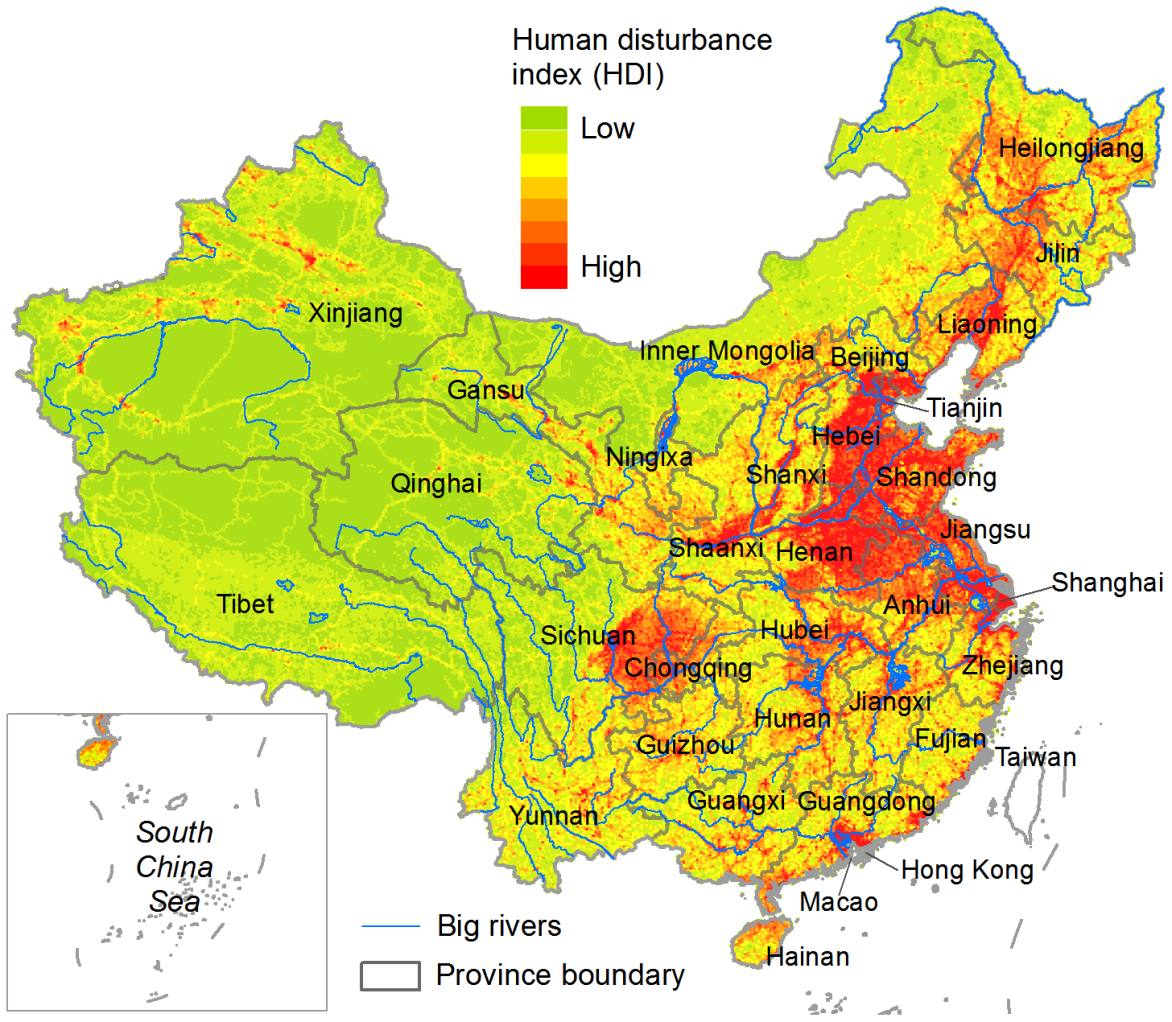
Human disturbance index (HDI). HDI was modeled by aggregating information on several socioeconomic proxies, including proportion of land converted by human use, human population density, gross domestic product, and road density.

We used several socioeconomic proxies, including proportion of land converted by human use, human population density, gross domestic product (GDP) and road density to calculate the human disturbance index (HDI) or human footprint [4], [30]. The basic planning units were regular hexagons, each sized 100 km^2^. The analysis included three steps. First, we calculated an individual HDI (IHDI) for each of these proxies. For proportion of converted land, we calculated the IHDI as the percent area of human-developed-land use – including croplands, plantations, rural settlements and urban/industrial areas – within each hexagon. For human population density and GDP, we calculated the IHDIs as their mean values per square kilometer within each unit. For road density, we considered four transportation levels (i.e., railway, expressway, national-provincial road and other-level roads), and calculated an IHDI for each level as the total road length within each unit. Second, we normalized the data ranges of all IHDIs on a scale of from 0.00 to 1.00, and then summed them to get the total HDI. Finally, we empirically transformed the data range of the total HDI on a scale of from 10.00 to 300.00 (Figure 1) so as to clearly demonstrate the overall human disturbance pattern.

We obtained datasets on land uses, human population density and GDP from the Data Center for Resources and Environmental Sciences of the Chinese Academy of Sciences [31], and all are 1 km×1 km resolution grid files. The road networks were derived from the national 1∶ 1,000,000 geographic database.

### Conservation Priority-setting

We used the software Marxan (v2.0.2) to implement the conservation priority-setting process. Marxan was developed to cost-effectively solve an optimization problem of representing a suite of biodiversity targets [17]. To ensure that all conservation features were captured across their ranges of environmental and genetic variations [32], we first stratified their ranges with China's 53 terrestrial ecoregions [33], and then defined a quantitative conservation target for each feature per ecoregion. Due to limited data available for setting up appropriate conservation targets [34], we defined the target for each conservation feature as a uniform percentage area of its distribution range as suggested in previous studies (e.g., [3], [32]). Specifically, the quantitative targets were selected based on expert opinions as follows: 30% for endangered species, 20% for priority natural ecosystems and 10% for natural vegetation types. An internationally recognized lowest target of 10% was set for natural vegetation types because they were assumed to represent the variety of basic habitat types.

We ran Marxan with two scenarios – a HDI-considered scenario and a HDI-ignored scenario. For the HDI-considered scenario, we integrated HDI values as a penalty function in Marxan analysis, i.e. a unit having a higher degree of disturbance would receive a greater penalty. For the HDI-ignored scenario, we used a uniform penalty of 1.0 per unit. The units with greater HDI values exhibit a more highly degraded ecological condition and should offer less potential from a conservation perspective [4]. Therefore, Marxan's algorithm sought to identify the optimized priority areas by minimizing the total HDI score in the HDI-considered scenario or the total land area in the HDI-ignored scenario. For the Marxan configurations, we: (1) generated 1000 solutions; (2) included a boundary length file and a modifier factor to control the compactness of priority areas; (3) implemented Simulated Annealing followed by Iterative Improvement; and (4) used the default values for Number of Iterations (1,000,000) and Temperature Decreases (10,000).

We derived the conservation value that reflects the relative priority or irreplaceability of each planning unit [3] from the frequency of solutions selected, and used the best of the 1,000 solutions as the most cost-effective portfolio of priority units. We then identified the irreplaceable units as those selected in more than 800 solutions; 80% is often used for accuracy assessment for spatial data (e.g., [35]).

### Effects of Human Disturbance

We compared the total HDI score and total area of the two portfolios of priority units generated by the HDI-ignored and HDI-considered scenarios, respectively. The changes in irreplaceable areas between the two scenarios were assessed in terms of the total area and the proportional area changes by province.

To assess the effects of human disturbance at a finer-scale, we further investigated the distributions of the priority units and irreplaceable areas on different environmental zones of HDI, elevation, and terrain ruggedness. We derived seven zones for each variable as follows: (1) We classified HDI zones by applying the Quantile Classification Scheme on HDI values; (2) We derived elevation zones from the SRTM 90 m DEM according to studies on geomorphology [36] (the elevation classification schemes were <200, 200–500, 500–1,000, 1,000–1,500, 1,500–2,000, 2,000–4,000, and >4,000 m); (3) We calculated a terrain ruggedness index (TRI) as the average difference in elevation between a center cell and its eight neighboring cells using the SRTM DEM, and the Quantile Classification Scheme was then used to break the TRI values into seven terrain categories, i.e. level, near-level, slightly-rugged, intermediately-rugged, moderately-rugged, highly-rugged and extremely-rugged [37]–[38].

### Spatial Patterns of Conservation Priorities

Using the outputs from the HDI-considered scenario, we analyzed the spatial distributions of priority units and irreplaceable areas on HDI, elevation, TRI zones and provinces. We then delineated the primary large-scale priority areas as the large clusters of high-conservation-value planning units through an expert-based visual interpretation process.

## Results

### Effects of Human Disturbance

**Figure 2:**
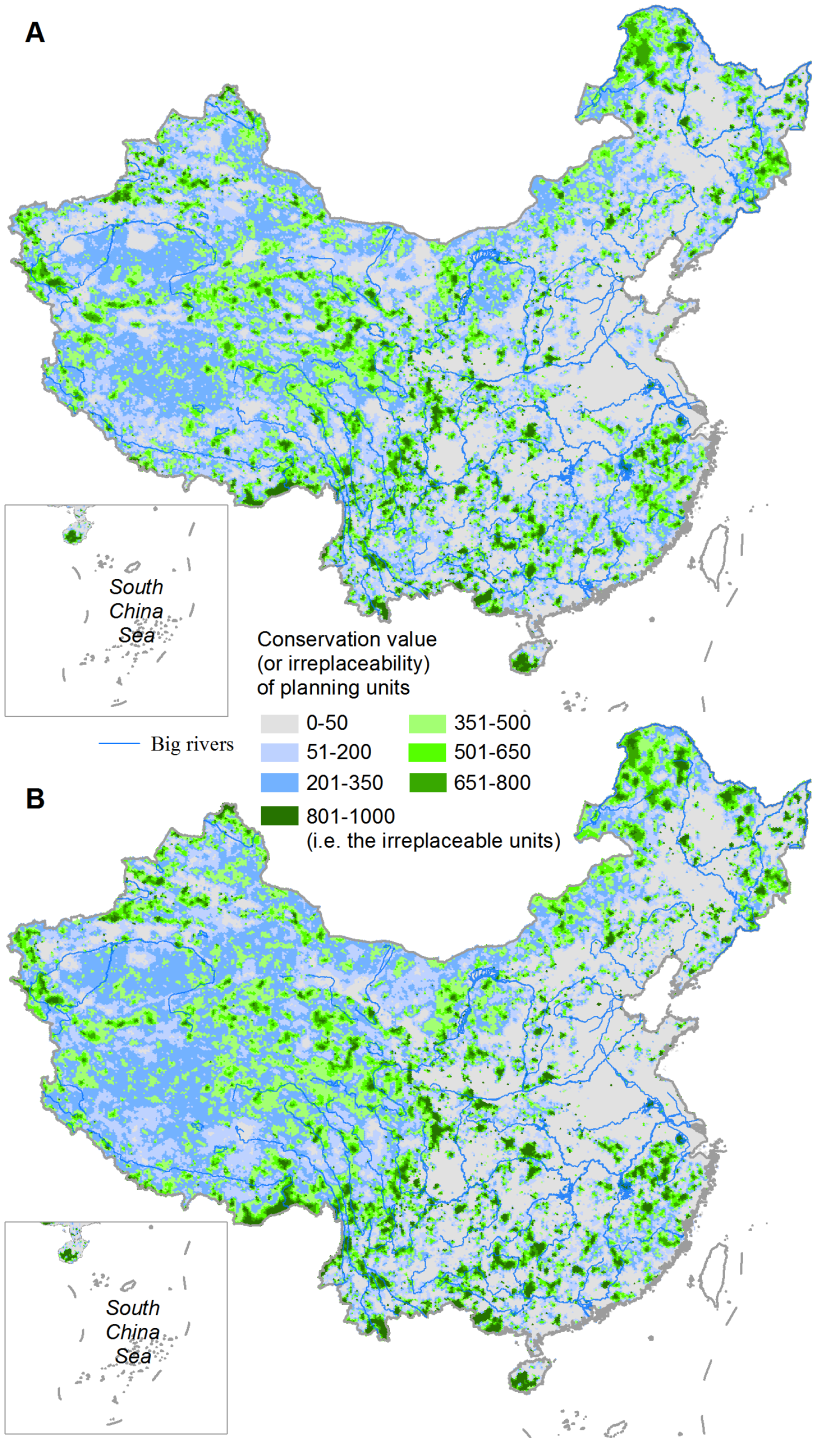
The conservation value of 100^2^ hexagon units for achieving the defined conservation targets. (A) HDI-ignored scenario and (B) HDI-considered scenario.

**Figure 3:**
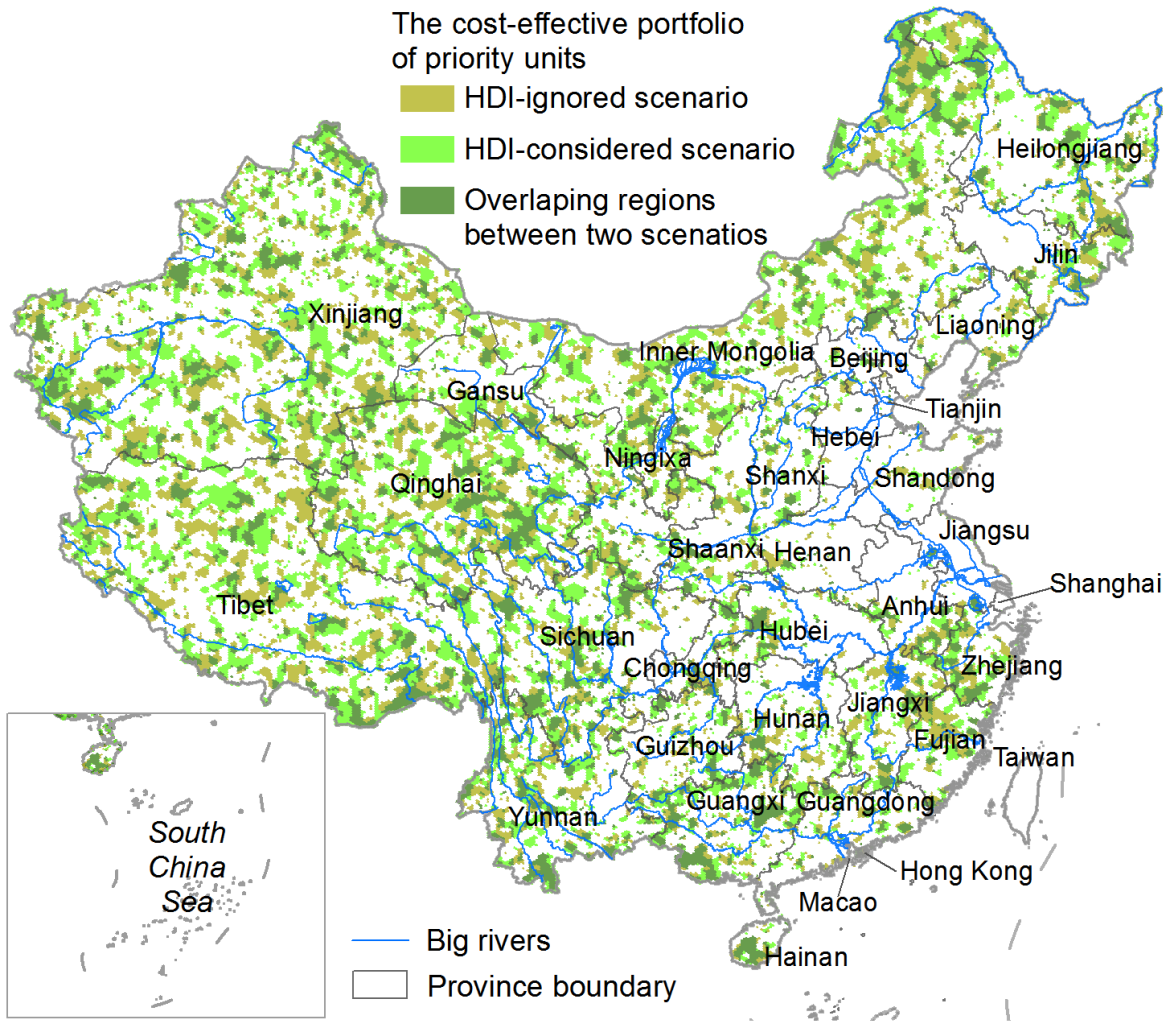
The cost-effective portfolios of priority units identified by the HDI-ignored and HDI-considered scenarios, respectively.

We presented the conservation value (based on a scale of from 0 to 1,000) of individual 100 km^2^ hexagon units distributed throughout China (Figure 2). The portfolio of priority units in the HDI-ignored scenario (Figure 3) covered 24.6% of China's land area. By explicitly including the HDI as an additional penalty, we achieved the same conservation targets with a small increase (∼7%) in the total area of priority units compared to when HDI was ignored, meanwhile a clear reduction of ∼9% in the total HDI score was observed. The overlapping region (Figure 3) covered 46.3% and 43.2% of the priority units in the HDI-ignored and HDI-considered scenarios, respectively. A strong and positive spatial correlation exists (Spearman's rank correlation, *r* = 0.871, *p*<<0.001) between the two conservation value layers.

**Figure 4:**
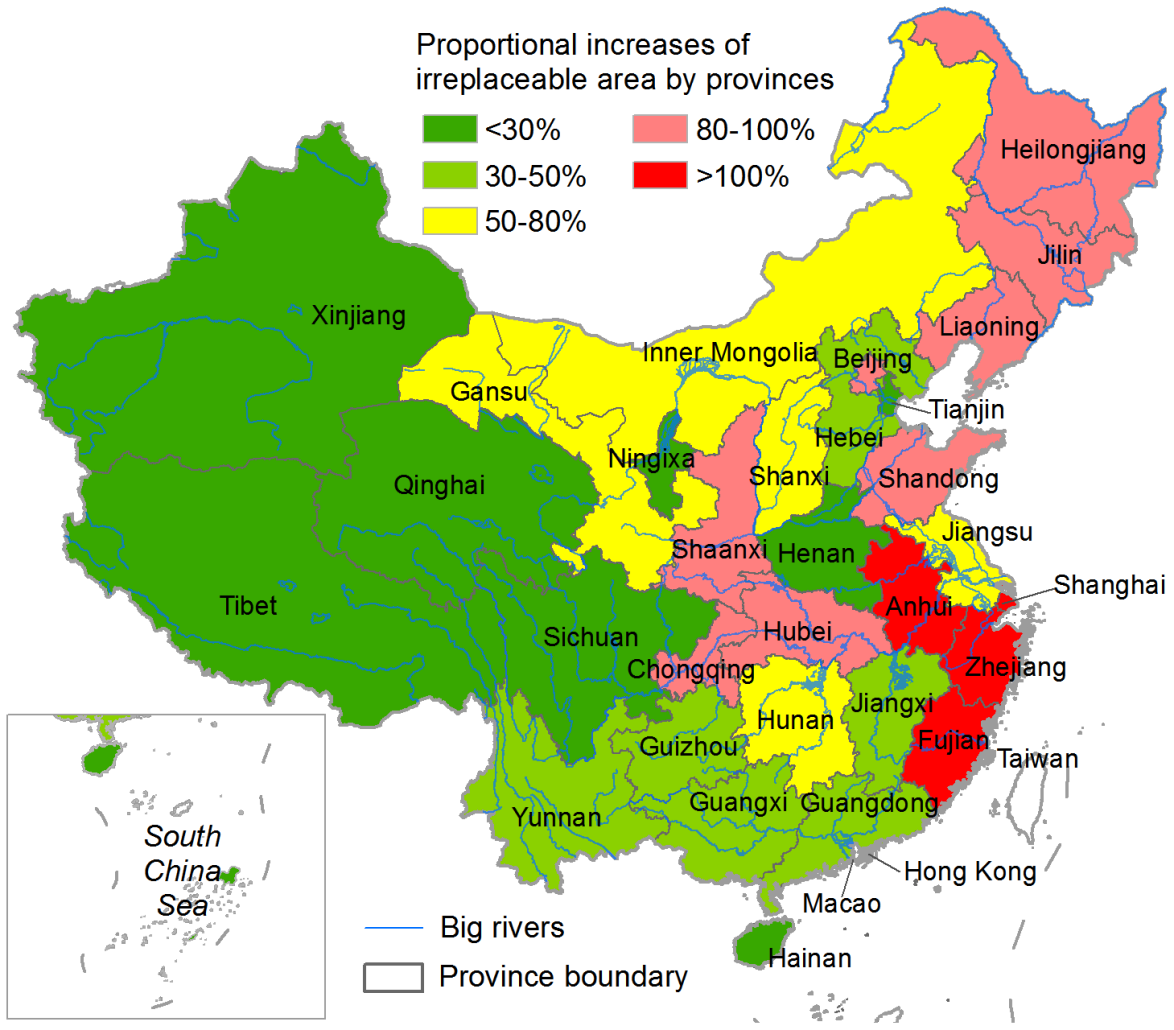
Proportional changes in irreplaceable area between the HDI-ignored and HDI-considered scenarios by province.

The irreplaceable units in the HDI-ignored scenario (Figure 2A) covered 2.8% of China's landmass, while an increase of ∼43% in the total irreplaceable area was observed in the HDI-considered scenario (Figure 2B). The overlapping region occupied 82.7% and 57.7% of the irreplaceable areas in the HDI-ignored and HDI-considered scenarios, respectively. High proportional increases in irreplaceable area occurred principally in provinces located in the eastern coastal region, middle-lower Yangtze River Basin and northeastern China, whereas provinces in western and southwestern China had the fewest changes (Figure 4). Several provinces in the eastern highly-disturbed regions (Figure 1), including Guangdong, Jiangxi, Henan and Hebei, also were found to have small changes in their irreplaceable areas (Figure 4).

**Figure 5:**
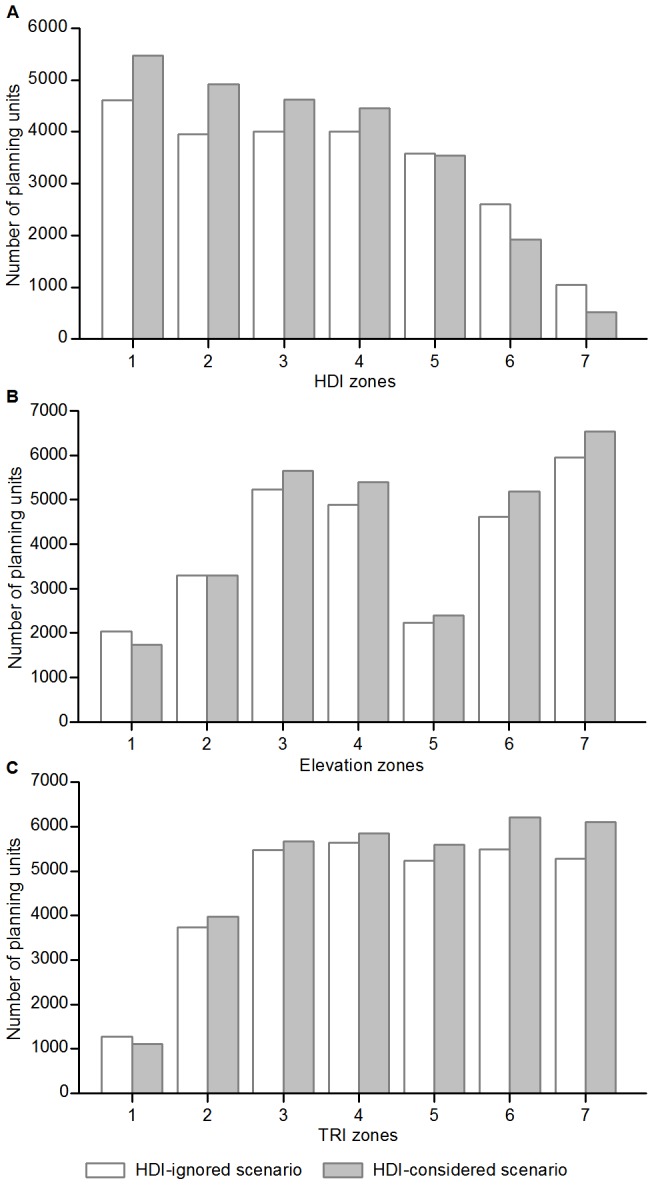
The distribution of priority units on (A) HDI, (B) elevation, and (C) TRI zones. The numbers 1 to 7 on the horizontal axes represent (A) low to high HDI value classifications, (B) elevation zones of <200, 200–500, 500–1,000, 1,000–1,500, 1,500–2,000, 2,000–4,000, and >4,000 m, and (C) terrain categories of level, near-level, slightly-rugged, intermediately-rugged, moderately-rugged, highly-rugged, and extremely-rugged.

**Figure 6:**
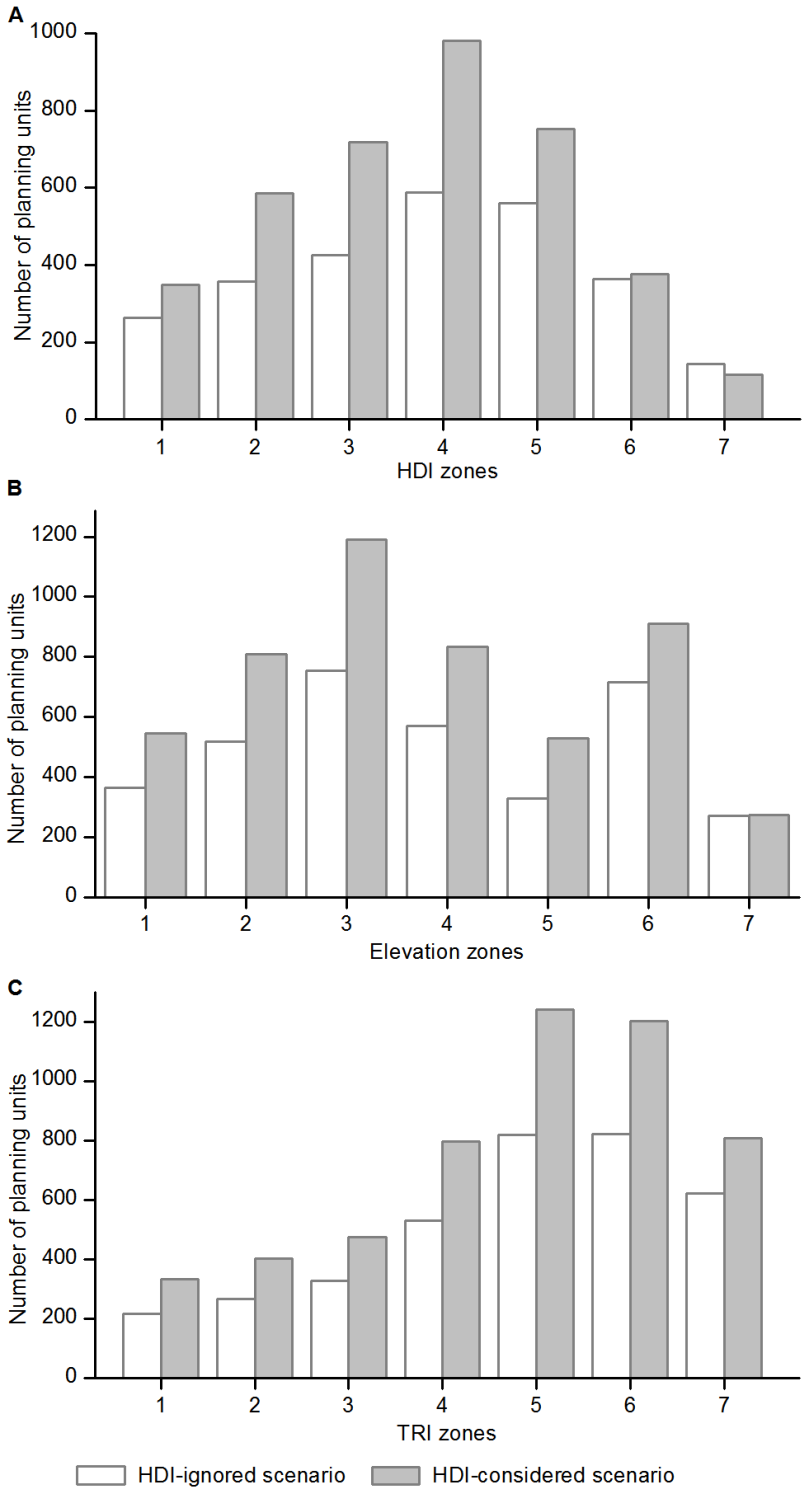
The distribution of irreplaceable units on (A) HDI, (B) elevation, and (C) TRI zones. See Figure 5 for the explanation of numbers 1 to 7 on the horizontal axes.

Compared to the results in the HDI-ignored scenario, the portfolio of priority units in the HDI-considered scenario contained: (1) fewer units in the three highest HDI zones and more units in the four lower HDI zones, (2) fewer units only in the lowest (<200 m) elevation zone and more units in the other six zones, and (3) fewer units in the level TRI zone and more units in each of the other TRI zones (Figure 5). The HDI-considered scenario identified a greater number of irreplaceable units in almost all environmental zones than did the HDI-ignored scenario, with the sole exception of the highest HDI zone (Figure 6).

### Spatial Patterns of Conservation Priorities

We analyzed the spatial patterns of conservation priorities using the outcomes from the HDI-considered scenario. The priority units consistently decreased with increasing HDI value (Figure 5A), with the majority (∼76%) located in the four lower HDI zones. The <200 m elevation zone included only 5.8% of all priority units, and the zones of 200–1,000, 1,000–2,000 and >2,000 m contained 29.6%, 25.8% and 38.8% of the priority units, respectively. The priority units generally had an increasing distribution trend on TRI zones from level to extremely-rugged terrain (Figure 5C), with the vast majority located in slightly- to extremely-rugged zones, and only 3.2% identified in level zone and 11.5% in near-level zone. All provinces included some units that were required for meeting the conservation targets (Figure 3), with the greatest proportion occurring in Xinjiang followed by Tibet, Inner Mongolia, Qinghai, Sichuan and Yunnan. These six western provinces contained 72.5% of the total priority units.

The irreplaceable units had a normal-like distribution on the HDI zones that peaked in the fourth zone (Figure 6A). Compared to the distribution of priority units, greater proportions of irreplaceable units were selected in lower elevation zones, with 10.7%, 39.3%, 26.7% and 23.3% of the total irreplaceable area located at <200, 200–1,000, 1,000–2,000 and >2,000 m zones, respectively. In particular, the highest zone (>4,000 m) contained the smallest proportion of irreplaceable areas (Figure 6B) although the greatest number of priority units occurred there (Figure 5B). In addition, over 75% of the irreplaceable areas were located in intermediately- to extremely-rugged TRI zones (Figure 6C). Provinces with the greatest number of irreplaceable areas were Yunnan followed by Guangxi, Tibet, Xinjiang, Inner Mongolia and Sichuan, and they contained 51.5% of the total irreplaceable area.

**Figure 7:**
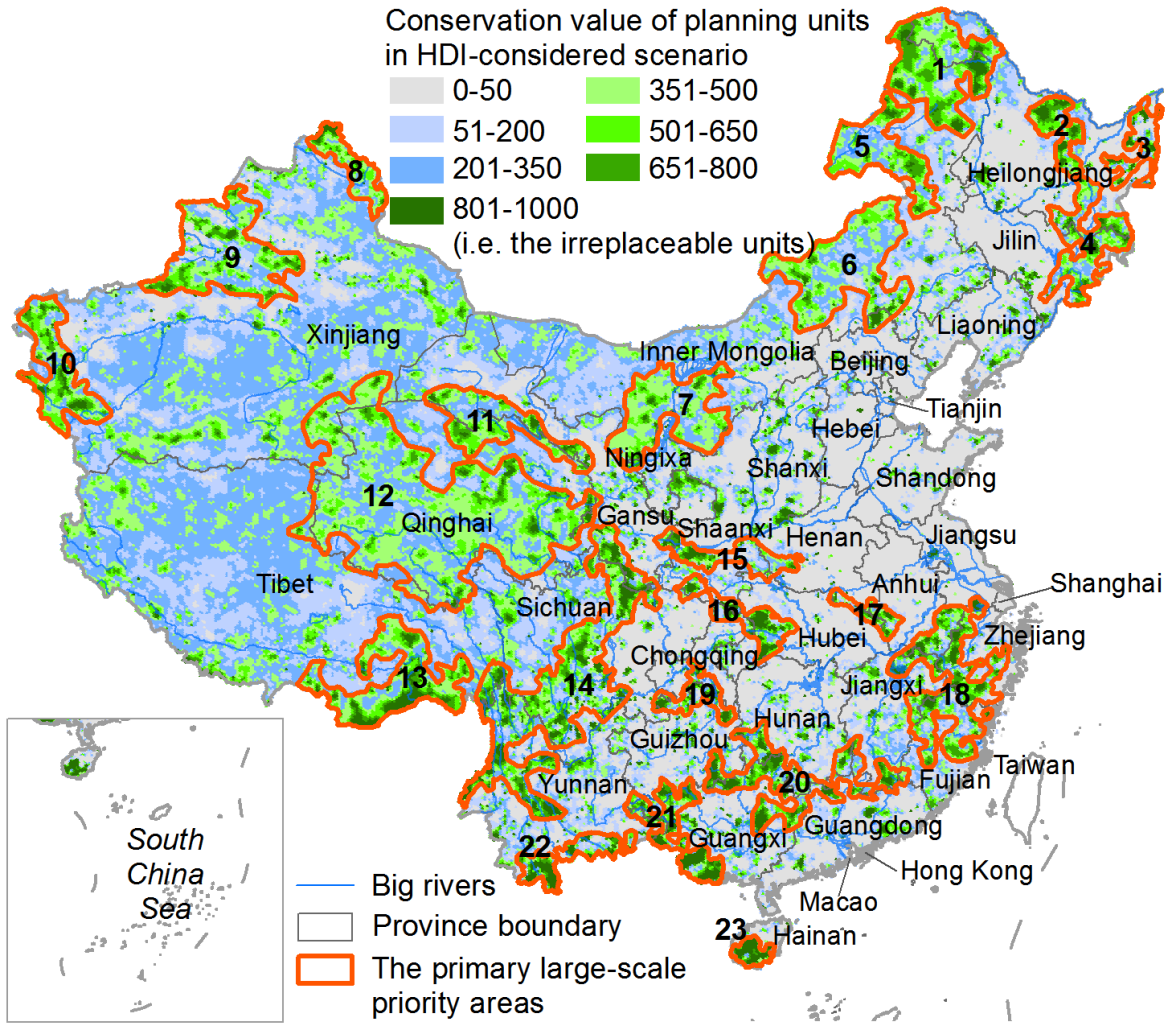
The distribution of the 23 primary large-scale priority areas. 1 – Daxing'anling Mountain, 2 – Xiaoxing'anling Mountain, 3 – Sanjiang Plain, 4 – Changbai Mountain, 5 – Hulunbuir Grassland, 6 – Xilingol Grassland, 7 – Alashan-Ordos Region, 8 – Altai Mountain, 9 – Tianshan Mountain, 10 – Pamirs Plateau, 11 – Qilian Mountain, 12 – Sanjiangyuan-Qiangtang Region, 13 – Southeast Himalaya Mountain, 14 – Hengduan Mountain, 15 – Qinling Mountain, 16 – Daba Mountain, 17 – Dabieshan Mountain, 18 – Mountain Region connecting Fujian-Zhejiang-Jiangxi-Anhui, 19 – Wuling Mountain, 20 – Nanling Mountain, 21 – Mountain Region in western Guangxi, 22 – Xishuangbanna, and 23 – Southern Hainan Island.

Overall, many more units in western China were assigned higher conservation values compared to eastern and southern regions, where the distributions of high-value units were severely fragmented (Figure 7). Based on the conservation value data and expert knowledge, we visually delineated the boundaries of 23 primary large-scale priority areas and excluded many small isolated areas (Figure 7). These large-scale priority areas covered ∼28% of China's landmass and were mainly distributed in remote regions at high elevation and/or rugged terrain. Regions that have experienced high-intensity disturbances, e.g. Northeast China Plain, North China Plain, South Huaihe and Middle-lower Yangtze River Plain, Sichuan Basin and Pearl River Delta Area, did not contain any large-scale priority areas (Figure 7).

## Discussion

In this study we implement a rigorous planning framework to identify the optimized national-scale conservation priorities in China. Our framework addresses several key features overlooked in previous studies, i.e. human disturbance, integration of ecosystem- and species-level conservation features, and principles of complementarity and representativeness.

### Effects of Human Disturbance

Due to a lack of site-specific data on the ecological integrity of most biodiversity features [16], a HDI (or suitability index) is often modeled by aggregating human disturbance data to provide an indirect measure of ecological condition [4], [30]. By explicitly considering HDI, our goal is to direct conservation towards the least-disturbed regions while still fully meeting conservation goals. We feel that this approach will promote conservation success and more efficiently achieve conservation goals [3], [6]. Moreover, areas with higher disturbances offer less conservation potential as they have lower habitat suitability for sustaining conservation features [4].

Our result indicates that the portfolio of priority units in the HDI-considered scenario is characterized by a marked reduction in the total HDI score and a slight increase in the total area, and in addition, more priority units are identified at less-disturbed, higher and/or rugged regions (Figure 5). Such effects are derived from implementing Marxan's algorithm for identifying an optimized portfolio of priority areas that has the minimum total penalty score [17]. Therefore, many priority units identified in the HDI-ignored scenario, especially those distributed as fragments on highly-disturbed lands, were excluded or devalued in the HDI-considered scenario so as to minimize the total HDI score of the portfolio. This requires the HDI-considered scenario to select a greater number of priority units with lower HDI values to achieve the same conservation goals, because each of these units contains relatively fewer conservation features and/or covers smaller areas within their distribution ranges.

The total irreplaceable area in the HDI-considered scenario increased significantly (∼43%) and more irreplaceable units were selected in almost all HDI, elevation, and TRI zones except the highest HDI zone (Figure 6). We think the increase results from the fact that Marxan solutions favor those units with relatively lower penalty scores, which also was reported by Carwardine et al. [3]. This indicates that human disturbance can partly degrade the potential options available for implementing cost-effective conservation. Our result, that the most highly-developed provinces had the greatest proportional increases in irreplaceable area while western less-disturbed provinces had smaller changes (Figure 4), also supports this perspective. However, we also found that several highly-developed provinces had only small changes in irreplaceable area. We think this is because those provinces contain relatively fewer conservation features and limited overlap exists between the distributions of conservation features and areas of human disturbances.

A fundamental concern in including human disturbance is that priority areas may be biased to remote, higher and more rugged places. Such a biased distribution has been a severe problem resulting in the existing reserve networks failing to adequately represent the overall biodiversity [34], [38]. Does our analysis further increase the existing biases in the location of established reserves? We feel it does not, because our framework implements ‘representativeness’ as a core principle in identifying priority areas and defines explicit conservation targets for all selected conservation features. The goal for representing the full range of biodiversity requires that the priority-setting process also focuses on disturbed landscapes of high biodiversity conservation significance [5]. Similar to Linke et al. [4], we integrated human disturbance as a discounting factor for ecological condition so as to ensure that the resulting portfolio was optimized for maximizing conservation achievements.

Although apparent shifts of priority units towards less-disturbed zones were observed (Figure 5A), the HDI-considered scenario only selected fewer priority units in the <200 m elevation zone (Figure 5B) and level zone (Figure 5C), and identified more irreplaceable units in almost all HDI, elevation, and TRI zones except the highest HDI zone (Figure 6). The lowest/level zone may provide less conservation potential because of limited current biodiversity in response to long-term human disturbance [33]. We also found considerable overlap, and strong and positive pairwise associations between the portfolios of priority units and the portfolios of irreplaceable areas identified by the HDI-ignored and HDI-considered scenarios, respectively. These results demonstrate that our analysis is conservation target based, and the inclusion of human disturbance did not result in the biased distribution of conservation priorities.

### Spatial Patterns of Conservation Priorities

Recognizing the advantages of including human disturbance in priority-setting, we analyzed the spatial patterns of conservation priorities using the results from the HDI-considered scenario. Human disturbance has caused severe degradation of natural ecosystems and many species extinctions, which can greatly diminish the conservation value of a region that was historically rich in biodiversity [14], [33]. Therefore, the higher the disturbance intensity, the lower the proportion of priority units was allocated in a region (Figure 5A). Rugged terrain often serves as a natural barrier for human development, and these mountainous areas have become refuges for many endangered species; These areas also are preferred as conservation priorities because they maintain more diverse habitats and higher animal and plant biodiversity [14].

We found higher percentages of irreplaceable area occurred in lower elevation zones (Figure 6B) compared to the distribution of priority units (Figure 5B). This implies that there are relatively fewer cost-effective options for fulfilling conservation targets in lowland regions, whereas the highland areas have greater flexibility in priority-setting. As moderately-disturbed and/or intermediately- to extremely-rugged zones contain the majority of irreplaceable areas (Figure 6), these habitats should be targeted to identify potentially important areas for implementing cost-effective conservation. These habitats are mainly found in western provinces, which include the vast majority of both priority units and irreplaceable areas, and therefore we consider those provinces to be of great significance in conserving China's biodiversity.

Previous researches have revealed that the remaining natural landscapes in eastern and southern China are highly fragmented, and western China supports more intact natural ecosystems and endangered species [14], [33]. This study similarly found that western China contains more high-value units clustered in relatively larger patches, while the high-value units in eastern and southern regions are severely fragmented and principally located in mountainous areas (Figure 7). Our result shows that the primary large-scale priority areas are mainly distributed in remote places with high elevation and rugged terrain (Figure 7). This finding is generally consistent with the results in previous studies (e.g., [10], [12]–[15]).

However, we also identified several priority areas that were rarely considered before, including the Hulunbuir Grassland, Xilingol Grassland, Alashan-Ordos Region, Altai Mountain, and Pamirs Plateau (Figure 7). All are located in Inner Mongolia and Xinjiang, covering grassland, semi-desert, alpine and tundra biomes. These areas are not rich in species diversity, but they are valued for maintaining several important ecosystems that sustain many endemic species and critical ecosystem services [39]. Our result agrees with the limited number of studies that have considered goals for ecosystem conservation. For example, the Alashan-Ordos Region and Altai Mountain are recognized as the key areas for protecting priority terrestrial ecosystems [39], and each of these five areas exhibits some overlap with the global 200 priority ecoregions, including Daurian/Mongolian Steppe, Altai-Sayan Montane Forests, and Middle Asian Montane Woodlands and Steppe [40].

The primary large-scale priority areas are the centers of biodiversity and evolution as they provide refuges for many species and sustain important ecosystem services [8], [12], and in these areas it is usually simpler and less expensive to implement conservation actions. In addition to establishing reserves, these areas should be subject to a variety of sustainable management approaches that seek to balance extractive uses with the retention of natural resources and ecosystem functions, such as the various ecosystem service policies currently implemented in China [9]. However, these large-scale priority areas are not sufficient to fulfill China's overall conservation goals [15], because many species, particularly those that exploit special microhabitats, may only occur in places close to developed landscapes and are already highly threatened [5]. Therefore, the priority units distributed in highly-disturbed regions that are not included in the large-scale priority areas should be included in local conservation actions. This may be even more urgent in order to prevent the immediate loss of biodiversity [41].

### The Priority-setting Framework

Using this priority-setting framework, we are trying to ensure the identification of a comprehensive and cost-effective portfolio of conservation priorities for China. The process is driven by explicitly delineating spatial distributions and quantitatively defined targets for representative conservation features. We believe the analysis is rigorous, objective, transparent, and replicable.

We acknowledge that the availability and accuracy of spatial data on biodiversity and disturbances are a primary constraint for national-scale priority-setting. Therefore, our results can be further refined as more comprehensive data become available. This study has used the most up-to-date national survey data on key protected plant and animal species [22], [25], as well as highly recognized information sources that have been used in previous studies (e.g., [14]). The ecosystem-level features represent meaningful biodiversity surrogates because they are the emergent entities of unique species assemblages and easily mapped; moreover, they are useful indicators of ecological processes and ecosystem services [16]. The integration of conservation features from different levels and multiple taxa can improve the effectiveness of priority areas in representing the overall biodiversity [5]. The HDI, a coarse simplification of current ecological condition, could be improved when better disturbance data and modeling methods are developed.

Hence we suggest that China increase its budget for improving the GIS-based conservation decision-making platform and enhance data sharing mechanisms. Moreover, the integration of ecological processes, ecosystem services, socioeconomic objectives and climate projections represents future research priorities in SCP [4], [6], [7].

### Application

Systematic conservation priority-setting has significant implications in assisting China in achieving its cost-effective conservation goals as a megadiversity country. For instance, this approach has been applied in conservation priority-setting for China nationwide, and the work is a key component for developing the National Biodiversity Strategies and Action Plans (NBSAPs) [8]. China's Ministry of Environmental Protection requires all provinces, major river watersheds, and counties develop their Local Biodiversity Strategies and Action Plans (LBSAPs) [8]. Thus, we applied this priority-setting approach to come up with the first provincial LBSAPs for Sichuan [42], and now this approach is in high demand in China.

Not only is this approach useful in its direct application to conservation planning, but it also has important applicability for strategic land-use planning and sustainable development practices; e.g. the Ecological Function Regionalization and Major Function Oriented Zoning [43]–[44] could be further refined using our approach. Such planning seeks to optimize the spatial patterns of economic development and environment protection by investigating the synergies and trade-offs between their distributions [45]. Areas recognized as conservation priorities should be primarily preserved for sustaining biodiversity and ecosystem services. As China is now adopting a new paradigm of sustainable development by undertaking a transition from conventional industrialization to ecological civilization [46] numerous redlines on natural resources and environment management (e.g., the Key Ecological Function Regions and Development Prohibited/Restricted Zones) have been established to ensure the country's ecological security [47]. This priority-setting approach is of great significance for determining the spatially optimized conservation network or redlines for strategic land-use planning.

## Conclusions

This study presents optimized national-scale spatial priorities for biodiversity conservation in China by implementing a systematic priority-setting approach with the integration of human disturbances, ecosystem- and species-level conservation features, and principles of complementarity and representativeness. Inclusion of human disturbance is essential for a cost-effective priority-setting – maximizing conservation achievement while minimizing conflicts with economic development. Such an approach will ensure the optimal spatial distribution of priority areas and reduce biases in conservation investment and/or land-use planning. The majority of priority units we identified are located in relatively remote, high and/or rugged places, however, areas that are moderately-disturbed, <2,000 m in altitude, and/or intermediately- to extremely-rugged in terrain should be targeted to identify potentially important regions for implementing cost-effective conservation. To achieve the overall biodiversity conservation goal in China, we delineate 23 primary large-scale priority areas, as well as recognize many isolated priority units in disturbed regions that need even more urgent conservation so as to prevent the immediate loss of biodiversity.

While requiring further refinement, our results provide valuable insights for current conservation and strategic land-use planning in China. This approach uses publicly available information, and is transferable and easy to implement by end-users, and applicable for national- and local-scale systematic conservation prioritization practices. Improved data, especially in the details of human disturbance and for aquatic systems at national-scale, will further enhance its applicability.

## References

[1] McCarthy DP, Donald PF, Scharlemann JPW, Buchanan GM, Balmford A, et al (2012) Financial costs of meeting global biodiversity conservation targets: current spending and unmet needs. Science 338: 946–949. 23065904 10.1126/science.1229803

[2] Brooks TM, Mittermeier RA, da Fonseca GAB, Gerlach J, Hoffmann M, et al (2006) Global biodiversity conservation priorities. Science 313: 58–61. 16825561 10.1126/science.1127609

[3] Carwardine J, Wilson KA, Ceballos G, Ehrlich PR, Naidoo R, et al (2008) Cost-effective priorities for global mammal conservation. Proceedings of the National Academy of Sciences of USA 105: 11446–11450. 10.1073/pnas.0707157105 PMC2495010 18678892

[4] Linke S, Kennard MJ, Hermoso V, Olden JD, Stein J, et al (2012) Merging connectivity rules and large-scale condition assessment improves conservation adequacy in river systems. Journal of Applied Ecology 49: 1036–1045.

[5] Margules CR, Pressey RL (2000) Systematic conservation planning. Nature 405: 243–253. 10821285 10.1038/35012251

[6] Venter O, Possingham HP, Hovani L, Dewi S, Griscom B, et al (2013) Using systematic conservation planning to minimize REDD+ conflict with agriculture and logging in the tropics. Conservation Letters 6: 116–124.

[7] Withey JC, Lawler JJ, Polasky S, Plantinga AJ, Nelson EJ, et al (2012) Maximising return on conservation investment in the conterminous USA. Ecology Letters 15: 1249–1256. 22913646 10.1111/j.1461-0248.2012.01847.x

[8] The Ministry of Environmental Protection of China (2011) China national biodiversity conservation strategy and action plans 2011–2030. Beijing: Chinese Environmental Science Press.

[9] Liu JG, Ouyang ZY, Yang W, Xu WH, Li SX (2013) Evaluation of ecosystem service policies from biophysical and social perspectives: the case of China. In: Levin SA, editor. Encyclopedia of biodiversity, second edition, volume 3. pp. 372–384. Waltham: Academic Press.

[10] Chen LZ (1993) China's biodiversity and conservation strategy. Beijing: Science Press.

[11] Ma KP (2001) Hotspots assessment and conservation priorities identification of biodiversity in China should be emphasized. Acta Phytoecologica Sinica 25: 125.

[12] Chen CD (1998) Biodiversity of China: a country study. Beijing: Chinese Environmental Science Press.

[13] Li DQ, Song YL, Ouyang ZY (2003) Research on the national forestry nature reserve system plan. Beijing: China Land Press.

[14] Tang ZY, Wang ZH, Zheng CY, Fang JY (2006) Biodiversity in China's mountains. Frontiers in Ecology and the Environment 4: 347–352.

[15] Zhang YB, Ma KP (2008) Geographic distribution patterns and status assessment of threatened plants in China. Biodiversity and Conservation 17: 1783–1798.

[16] Groves CR (2003) Drafting a conservation blueprint: a practitioner's guide to planning for biodiversity. Washington DC: Island Press.

[17] Ardon JA, Possingham HP, Klein CJ (2010) Marxan good practices handbook, Version 2. Victoria: Pacific Marine Analysis and Research Association.

[18] Crous CJ, Samways MJ, Pryke JS (2013) Exploring the mesofilter as a novel operational scale in conservation planning. Journal of Applied Ecology 50: 205–214.

[19] Zhang XS (2007) Vegetation map of People's Republic of China (1: 1,000,000). Beijing: Geology Press.

[20] IUCN (2012) The IUCN red list of threatened species. Available: http://www.iucnredlist.org. Accessed 10 November 2012.

[21] Fu LG (1992) China plant red data book: endangered and rare plants. Beijing: Science Press.

[22] State Forestry Administration of China (2009) National key protected wild plant resources survey. Beijing: China Forestry Publishing House.

[23] Institute of Botany (2009) Subject database of China plants. Available: http://www.plant.csdb.cn. Accessed 25 October 2012.

[24] WCS (2005) China species information services. Available: http://www.baohu.org. Accessed 20 May 2012.

[25] State Forestry Administration of China (2009) National key terrestrial wildlife resources survey. Beijing: China Forestry Publishing House.

[26] Institute of Zoology (2009) Database of Fauna Sinica. Available: http://www.zoology.nsdc.cn. Accessed 15 September 2012.

[27] Zhang RZ (1997) Distributions of China mammal species. Beijing: China Forestry Publishing House.

[28] Wang S (1998) China red data book of endangered animals: mammals. Beijing: Science Press.

[29] USGS (2004) Shuttle Radar Topography Mission DEM. Available: http://glcf.umiacs.umd.edu/data/srtm. Accessed 16 July 2012.

[30] Sanderson EW, Jaiteh M, Levy MA, Redford KH, Wannebo AV, et al (2002) The human footprint and the last of the wild. BioScience 52: 891–904.

[31] Yang XH, Ma HQ (2009) Natural environment suitability of China and its relationship with population distributions. International Journal of Environmental Research and Public Health 6: 3025–3039. 20049243 10.3390/ijerph6123025 PMC2800331

[32] Chan KMA, Shaw MR, Cameron DR, Underwood EC, Daily GC (2006) Conservation planning for ecosystem services. PLoS Biology 4: e379. 17076586 10.1371/journal.pbio.0040379 PMC1629036

[33] Wu RD, Zhang S, Yu DW, Zhao P, Li XH, et al (2011) Effectiveness of China's nature reserves in representing ecological diversity. Frontiers in Ecology and the Environment 9: 383–389.

[34] Rodrigues ASL, Andelman SJ, Bakarr MI, Boitani L, Brooks TM, et al (2004) Effectiveness of the global protected area network in representing species diversity. Nature 428: 640–643. 15071592 10.1038/nature02422

[35] Lea C, Curtis AC (2010) Thematic accuracy assessment procedures: National Park Service vegetation inventory, version 2.0. Natural resource report NPS/2010/NRR-2010/204. Fort Collins: National Park Service, US Department of the Interior.

[36] Li BY, Pan BT, Han JF (2008) Basic terrestrial geomorphological types in China and their circumscriptions. Quaternary Sciences 28: 535–543.

[37] Riley S, DeGloria SD, Elliot R (1999) A terrain ruggedness index that quantifies topographic heterogeneity. Intermountain Journal of Sciences 5: 23–27.

[38] Wu RD, Ma GZ, Long YC, Yu JH, Li SN, et al (2011) The performance of nature reserves in capturing the biological diversity on Hainan Island, China. Environmental Science and Pollution Research 18: 800–810. 21234808 10.1007/s11356-011-0440-5

[39] Xu WH, Ouyang ZY, Huang H, Wang XK, Miao H, et al (2006) Priority analysis on conserving China's terrestrial ecosystems. Acta Ecologica Sinica 26: 271–280.

[40] Olson DM, Dinerstein E (2002) The global 200: priority ecoregions for global conservation. Annals of the Missouri Botanical garden 89: 199–224.

[41] Joppa LN, Pfaff A (2009) High and far: biases in the location of protected areas. PLoS One 4: e8273. 20011603 10.1371/journal.pone.0008273 PMC2788247

[42] The Ministry of Environmental Protection of China (2011) Issue of Sichuan biodiversity conservation strategy and action plans. Available: http://www.zhb.gov.cn/zhxx/gzdt/201112/t20111215_221379.htm. Accessed 15 July 2013.

[43] The Ministry of Environmental Protection of China, Chinese Academy of Sciences (2008) Announcement on the issue of “National Ecological Function Regionalization”. Available: http://www.mep.gov.cn/info/bgw/bgg/200808/t20080801_126867.htm. Accessed 15 May 2013.

[44] The State Council of China (2011) Circular of the State Council on the issue of “Major Function Oriented Zoning”. http://www.gov.cn/zwgk/2011-06/08/content_1879180.htm. Accessed 20 May 2013.

[45] Fan J, Li P (2009) The scientific foundation of Major Function Oriented Zoning in China. Journal of Geographical Sciences 19: 515–531.

[46] Pan J (2012) From industrial toward ecological in China. Science 336: 1397. 10.1126/science.1224009 22700916

[47] Lü Y, Ma Z, Zhang L, Fu B, Gao G (2013) Redlines for the greening of China. Environmental Science & Policy 33: 346–353.

